# Désarticulation scapulo-thoracique, une conséquence d’un échec de traitement traditionnel chez une adolescente: à propos d’un cas

**DOI:** 10.11604/pamj.2023.46.49.41650

**Published:** 2023-10-03

**Authors:** Wendlamita Toussaint Tapsoba, Somkièta Modeste Francis Ouédraogo, Bernadette Béré, Saïd Nahl Oumar Ganamé, Thombiano Koundia, Oliver Zampou, Ouédraogo Isso, Bandré Emile

**Affiliations:** 1 Service de Chirurgie pédiatrique du Centre Hospitalier Universitaire Pédiatrique Charles de Gaulle de Ouagadougou, Ouagadougou, Burkina Faso

**Keywords:** Désarticulation, scapulo-thoracique, enfant, gangrène, cas clinique, Disarticulation, scapulo-thoracic, child, gangrene, case report

## Abstract

La désarticulation scapulo-thoracique pour une pathologie traumatique est exceptionnelle. La présentation de ce cas clinique vise à partager notre expérience à propos d'un cas de désarticulation scapulo-thoracique et situer l'intérêt d'une telle indication. Il s'est agi d'une patiente de 12 ans, de latéralité droite, scolarisée, qui a été hospitalisée pour traumatisme fermé du membre thoracique droit compliqué d'une infection après une semaine de traitement traditionnel (massage, bandage avec attelles en roseau). Le diagnostic de fracture du col chirurgical de l'humérus droit compliquée d'une gangrène humide de tout le membre et d'une anémie sévère, a été posé. Une transfusion sanguine, une triple antibiothérapie et une désarticulation scapulo-humérale ont été nécessaires. Une excision progressive de tissus nécrotiques puis une scapulectomie (à J24) pour nécrose scapulaire ont permis d'aseptiser le foyer et de réaliser une greffe cutanée (à J43). La patiente, sortie après 2 mois d'hospitalisation, avec un suivi conjoint en psychologie et en kinésithérapie, a présenté des suites opératoires simples sur un recul de 7 mois. L'échec du traitement traditionnel des fractures de membre peut mettre en jeu le pronostic vital et occasionner de graves séquelles.

## Abstract (en)

Scapulothoracic dissociation due to a trauma is exceptional. The purpose of this study was to share our experience with a case of scapulothoracic dissociation and to explain the advantages of our approach. We report the case of a 12-year-old right-handed schoolgirl admitted to hospital with a blunt trauma to the right thoracic limb complicated by infection after a week of traditional treatment (massage, bandaging with reed splints). Surgical neck fracture of the right humerus complicated by wet gangrene of the entire limb and severe anaemia was diagnosed. Blood transfusion, triple antibiotic therapy and scapulohumeral disarticulation were required. Progressive excision of necrotic tissue followed by scapulectomy (day 24) for scapular necrosis made it possible to sterilise the site and to perform a skin graft (day 43). The patient was discharged after 2 months. Physiotherapy was combined with psychological treatment and the patient had a simple post-operative course during the 7-month follow-up period. Failure of conventional treatment for limb fractures can be life-threatening and cause severe sequelae.

## Introduction

L'amputation de membre est un acte chirurgical mutilant, un ultime recours pour le chirurgien [1]. L'une des principales causes en Afrique est la gangrène consécutive à l'échec de traitement traditionnel des fractures de membres chez l'enfant [2]. La désarticulation scapulo-thoracique est rare et concerne principalement la pathologie cancéreuse [3,4]. Nous présentons un cas consécutif à une fracture humérale mal traitée chez une adolescente pour partager notre expérience et situer l'intérêt d'une telle indication.

## Patient et observation

**Présentation du patient:** il s'est agi d'une patiente de 12 ans, de latéralité droite, élève, qui a été hospitalisée pour traumatisme fermé du membre thoracique droit le 24 juillet 2014. Elle serait tombée du haut d'un arbre fruitier.

**Chronologie:** devant une douleur vive au bras droit avec une impotence fonctionnelle absolue du membre, elle avait été adressée à un rebouteux qui instaura des séances de massage avec un bandage sur des attelles en roseau. Après une semaine de traitement, devant la constatation d'un noircissement du membre et une fièvre, les parents ont consulté dans un centre médical d'où la patiente a été évacuée dans notre service. Elle ne présentait pas d'antécédent pathologique et n'aurait jamais fait l'objet d'une intervention chirurgicale.

**Résultats:** à l'admission, elle présentait une anémie et un sepsis sévères relatifs à une gangrène mixte de tout le membre thoracique droit.

**Figure 1:**
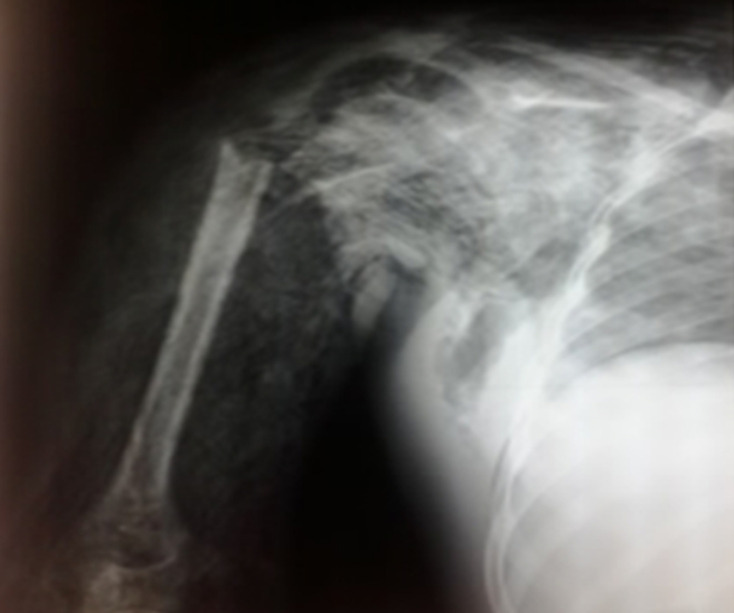
image radiographique pré-opératoire de la gangrène mixte du bras droit étendue à l'épaule après une fracture du col chirurgical de l'humérus

**Démarche diagnostique:** une radiographie avait montré une fracture du col chirurgical de l'humérus droit (Figure 1).

**Figure 2:**
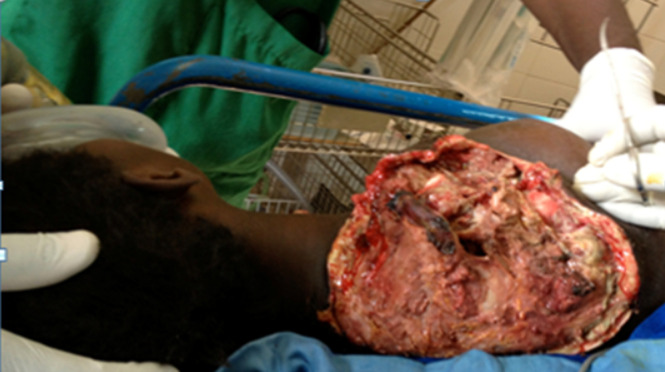
image post-opératoire de la désarticulation scapulo-humérale

**Figure 3:**
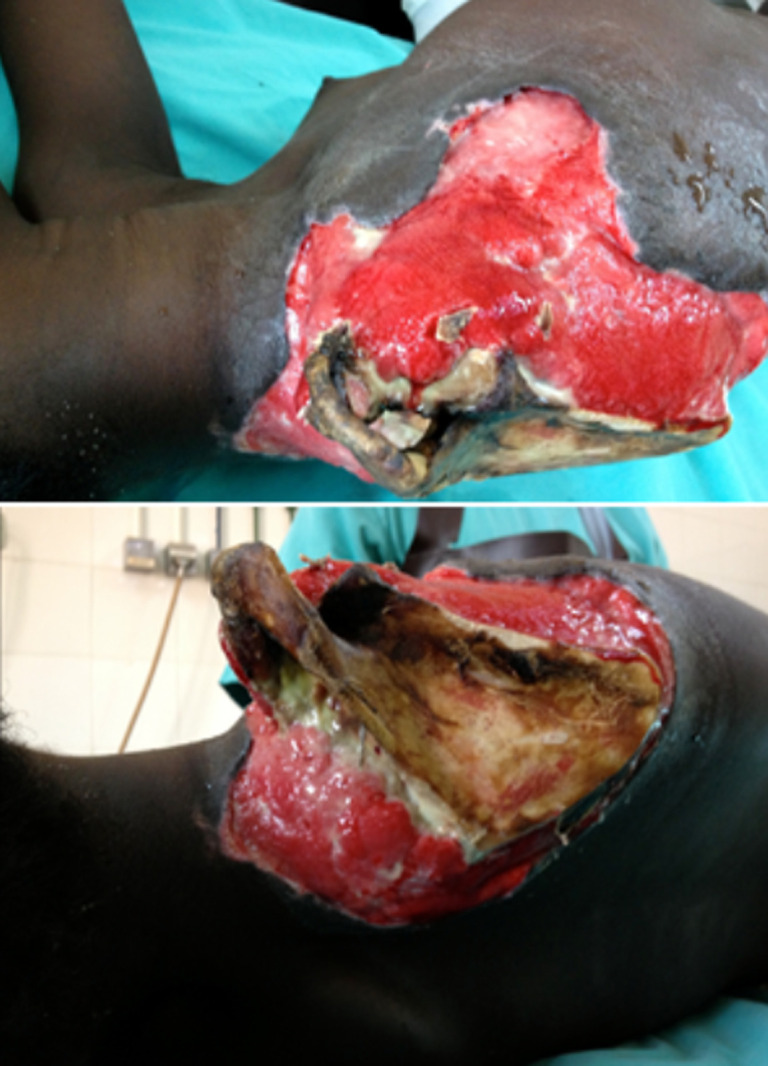
nécrose et dessiccation de la scapula et du 1/3 externe de la clavicule droite

**Interventions thérapeutiques:** une transfusion sanguine a été nécessaire. Une triple antibiothérapie et une désarticulation scapulo-humérale (Figure 2) ont permis de juguler l'infection. En per-opératoire, la ligature des vaisseaux a été réalisée en sub-clavière. La seconde étape chirurgicale était une excision progressive des tissus nécrotiques de l'épaule. Dans un 3^e^ temps, une scapulectomie associée à une résection du 1/3 latéral de la clavicule droite ont été réalisées en raison d'une nécrose osseuse et une dessiccation (Figure 3) à J24 post-opératoire; ce qui a permis d'aseptiser le foyer et de réaliser une greffe cutanée à J43.

**Figure 4:**
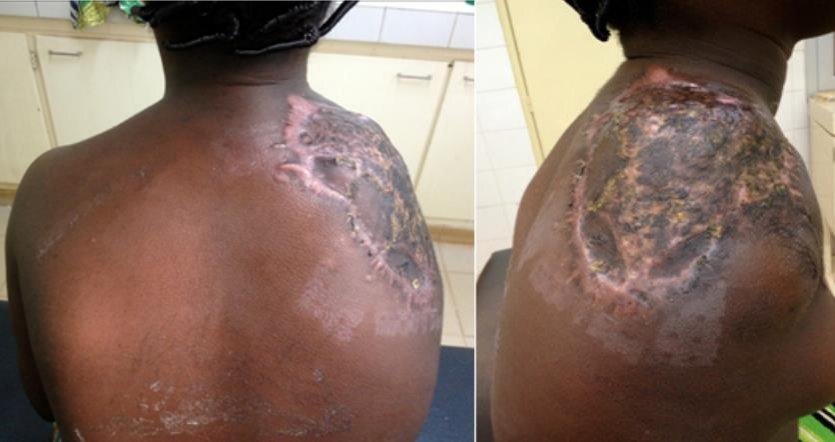
moignon de désarticulation scapulo-thoracique après greffe cutanée

**Suivi et résultat:** la patiente a été hospitalisée deux mois durant puis sortie après une cicatrisation primaire. Les suites opératoires étaient simples avec une bonne cicatrisation au contrôle clinique (Figure 4) à 7 mois après la sortie. Des suivis conjoints par une psychologue et une kinésithérapeute ont été instaurés pour l'aider à accepter son handicap physique et réintégrer son environnement social et scolaire.

**Consentement éclairé:** il a été obtenu.

## Discussion

Dans le cas clinique présenté, l'indication de la désarticulation scapulo-thoracique avait été posée devant la gangrène mixte extensive de tout le membre thoracique droit. Cette désarticulation a été réalisée en plusieurs étapes: désarticulation scapulo-humérale, excision progressive des tissus nécrotiques puis scapulectomie associée à une résection du 1/3 latéral de la clavicule. L'amputation doit être la plus économe possible sans compromettre le pronostic vital [4]. Dans ce contexte de nécrose tissulaire extensive avec suppuration, il était primordial de ligaturer les vaisseaux en zone saine pour éviter une dissémination septique. La ligature des vaisseaux huméraux chez cette patiente a été réalisée en sub-clavière. Les principales indications sont représentées essentiellement par les tumeurs osseuses malignes [3,4].

L'absence de moignon complique l'appareillage. La patiente sera contrainte à changer de latéralité pour les activités scolaires et de la vie courante. Des possibilités de prothèses de la scapula et du reste du membre thoracique existent mais non disponible et non accessible dans nos contrées en développement [2,4].

Ces séquelles à la fois fonctionnelles et esthétiques interpellent à œuvrer davantage pour la prévention des complications du traitement traditionnel des fractures de membre. Il serait difficile d'interdire cette pratique car nos populations y sont toujours attachées pour des raisons à la fois économiques et socio-culturelles [1,2,5]. L'expérience de certains pays a montré qu'une collaboration avec les rebouteux, notamment par une sensibilisation sur leurs limites et une réglementation de leur pratique, permet de réduire nettement le taux de complications [6,7].

## Conclusion

L'échec du traitement traditionnel des fractures de membre peut mettre en jeu le pronostic vital ou occasionner de graves séquelles fonctionnelles et esthétiques. La désarticulation scapulo-humérale est un geste chirurgical extrêmement mutilant; toutefois, dans le cas clinique présenté, elle a permis de donner un bon pronostic vital à la patiente devant la gangrène extensive de membre. Ces gangrènes de membre sont des complications évitables qui nécessiteraient une collaboration entre orthopédistes et rebouteux et une implication des responsables politiques dans nos pays en développement.
